# Analysis of fine-scale mammalian evolutionary breakpoints provides new insight into their relation to genome organisation

**DOI:** 10.1186/1471-2164-10-335

**Published:** 2009-07-24

**Authors:** Claire Lemaitre, Lamia Zaghloul, Marie-France Sagot, Christian Gautier, Alain Arneodo, Eric Tannier, Benjamin Audit

**Affiliations:** 1 Université de Bordeaux, Centre de Bioinformatique – Génomique Fonctionnelle Bordeaux, F-33000 Bordeaux, France; 2 Université de Lyon, F-69000 Lyon, France; 3 Laboratoire Biométrie et Biologie Evolutive, CNRS, Université Lyon 1, F-69100 Villeurbanne, France; 4 Équipe BAMBOO, INRIA Rhône-Alpes, 655 avenue de l'Europe, F-38330 Montbonnot Saint-Martin, France; 5 Laboratoire Joliot-Curie et Laboratoire de Physique, CNRS, Ecole Normale Supérieure de Lyon, F-69007 Lyon, France

## Abstract

**Background:**

The Intergenic Breakage Model, which is the current model of structural genome evolution, considers that evolutionary rearrangement breakages happen with a uniform propensity along the genome but are selected against in genes, their regulatory regions and in-between. However, a growing body of evidence shows that there exists regions along mammalian genomes that present a high susceptibility to breakage. We reconsidered this question taking advantage of a recently published methodology for the precise detection of rearrangement breakpoints based on pairwise genome comparisons.

**Results:**

We applied this methodology between the genome of human and those of five sequenced eutherian mammals which allowed us to delineate evolutionary breakpoint regions along the human genome with a finer resolution (median size 26.6 kb) than obtained before. We investigated the distribution of these breakpoints with respect to genome organisation into domains of different activity. In agreement with the Intergenic Breakage Model, we observed that breakpoints are under-represented in genes. Surprisingly however, the density of breakpoints in small intergenes (1 per Mb) appears significantly higher than in gene deserts (0.1 per Mb).

More generally, we found a heterogeneous distribution of breakpoints that follows the organisation of the genome into isochores (breakpoints are more frequent in GC-rich regions). We then discuss the hypothesis that regions with an enhanced susceptibility to breakage correspond to regions of high transcriptional activity and replication initiation.

**Conclusion:**

We propose a model to describe the heterogeneous distribution of evolutionary breakpoints along human chromosomes that combines natural selection and a mutational bias linked to local open chromatin state.

## Background

An important aspect of chromosome evolution is concerned with the maintenance of syntenies and with changes in the order of DNA fragments. Rearrangements that shuffle a chromosome organisation happen because of double-strand breaks and of the joining of double-stranded DNA ends, resulting in new conformations of the molecules. If the molecular bases of DNA damage and repair mechanisms that control these genetic instabilities at the cellular level are well described [1,2], the biophysical forces and selection constraints at work on an evolutionary timescale still need to be fully understood. For example, it remains mostly unexplained why the rate of chromosomal changes, estimated by the analysis of comparative maps between chicken and a number of mammals, is extremely variable (more than one order of magnitude of variation) both along evolutionary time and across different lineages [3,4]. In fact, despite recent progress made in this field of research, it remains an open question simply to design a model that would account for the observed positions of rearrangement breakpoints [5].

In 1984, Nadeau and Taylor [6] analysed the locations of 83 homologous loci between the human and mouse autosomes and observed that the length distribution of 13 conserved segments between these two genomes fits the distribution of a Poisson process, that is, rearrangement breakpoints are independently and uniformly distributed. This is referred to as the Random Breakage Model (RBM). Since then, the resolution of comparative maps has regularly improved. The number of delineated conserved segments between these two species increased by more than an order of magnitude [7-9]. This demonstrated the robustness of the main Nadeau and Taylor argument [10,11], at least for the large conserved chromosomal segments. The improved resolution also allowed to observe that the number of small conserved segments appears to be larger than predicted by the RBM [8,12-14], suggesting that the latter is not valid at a small scale (≲ 1 Mb). By comparing the number and size of breakpoints to the minimum number of rearrangements that can explain the structural differences between two genomes (each rearrangement event likely breaking the genome twice), Pevzner and Tesler [11] estimated that breakpoint regions are intensively reused in mammalian history. The validity of this argument has been criticised [15-18], further defended [19,20], and remains uncertain. Nevertheless, multi-species comparative maps pointed to the fact that, during bird and mammal evolution, breakpoints appear to have occurred in the same region over independent lineages more often than expected under the RBM [21-24]. Those breakpoint hotspots would then correspond to evolutionarily-stable fragile regions. The correlation between the localisations of breakpoints in these lineages and the distribution of several other genomic features such as segmental duplications [25,26], various repeated elements [27], experimental fragile sites [28,29], high GC content and CpG island density [24] provided further evidence suggesting that the distribution of breakpoint regions should be analysed in the context of a genome organisation. In this regard, Peng et al. [19] and Becker & Lenhard [5] proposed the Intergenic Breakage Model (IBM), where rearrangement breakages happen uniformly at random on the genome but are deleterious in genes, their regulatory regions, and in-between. The argument for the IBM is the simple fact that rearrangements that would break functional regions of the genome are selected against, which has since received some support from a functional analysis of vertebrate genomes [30]. If there seems to be strong evidence for the existence of some particular breakage hotspots, it remains unclear to which extent the model of evolution may deviate from the RBM, and what the alternative model would be.

Taking advantage of recent progress in the detection of rearrangement breakpoints in mammalian chromosomes that allowed us to locate them with a finer precision than obtained before [31], we propose some advances on the analysis of their distribution along the human genome relatively to the positions of genes and to the isochore organisation. Our results provide a direct comparison of the observed heterogeneous distribution of breakpoint regions with the predictions of the Intergenic Breakage Model, and bring new insight into the relationship between mammalian genome evolution and genome organisation. We further discuss these findings in terms of chromatin accessibility and elaborate on the necessity to take into account a mutational bias towards regions of replication initiation and of high transcriptional activity when deriving breakage models.

## Results

### Delineating mammalian evolutionary Breakpoint Regions

**Figure 1:**
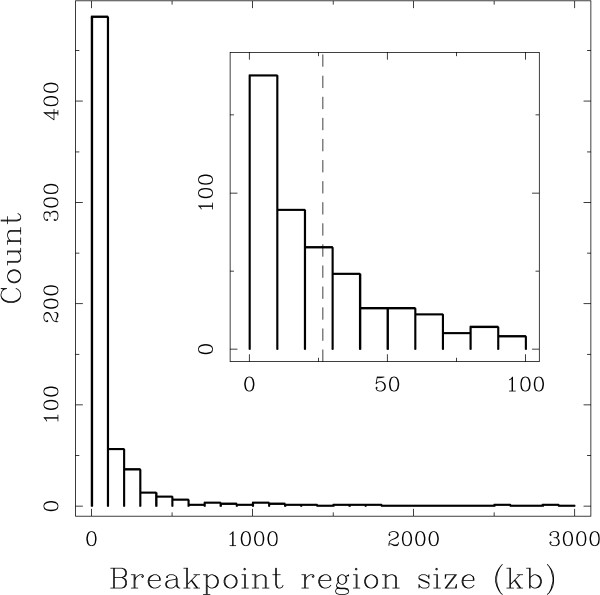
**Breakpoint region size distribution**. Histogram of BPR sizes (in kb) computed within classes of size 100 kb. (Inset) Same histogram limited to BPRs of size ≤ 100 kb computed in 10 kb classes; the vertical dashed line corresponds to the median BPR size (26.6 kb).

A Breakpoint Region (BPR) on the human genome is defined as a region that underwent at least one large chromosomal structural change, or is orthologous to such a region in a non-human lineage. We applied the method described in [31] to do pairwise comparisons between the genome of human and those of five other mammals (chimpanzee, macaque, mouse, rat, and dog). We thus obtained the positions on the human genome of all breakpoints differentiating the genome organisation of human from the genome organisation of each of the other five species. Mapping the whole set of BPRs from all lineages on the human genome, each group of intersecting BPRs was replaced by the intersection of all BPRs in the group when they could be explained by a unique rearrangement event, and by the union of all BPRs otherwise (see Methods). This procedure resulted in a data set of 622 non-intersecting BPRs with sizes spanning a wide range from 1 nucleotide (there is no ambiguity on the breakpoint location) to 2 887 673 nucleotides. The BPR size distribution is strongly skewed toward small values (Fig. 1): the BPR median size is 26.6 kb while 78% (483/622) of the BPRs have a length smaller than 100 kb. The BPRs are not uniformly distributed along the human chromosomes (Fig. S1 in Additional File 1) and this resolution allows us to compare their localisation with small scale genomic features such as genes.

Several rearrangements that break the DNA molecule at close loci may result in a single BPR because we may be missing some homologous markers between the breakpoints, or because intersecting BPRs from several lineages were grouped into one. In particular, large BPRs are more likely to contain several independent breakage events. To control for this effect in our analysis, every estimation of breakpoint density (see Methods) was performed with two modellings of the number of breakpoints within each BPR:

• Model M1: All BPRs contain exactly one breakpoint.

• Model M2: A BPR of length *L *(kb) contains a number of breakpoints *n *= max(1, *L*/100) i.e. when *L *≤ 100 kb, a BPR contains one breakpoint, while for *L *> 100 kb, BPRs are regions with 1 breakpoint per 100 kb.

Model M2 supposes that more than one breakpoint have aggregated (possibly in different lineages) inside large BPRs. The aggregation density parameter is not supposed to model the real number of breakages, but is meant to provide a control that the results are not biased due to an under-estimation of the number of breakage events in large BPRs. This choice of one breakage every 100 kb along large BPRs should be ample enough to test for this possible bias since it is one order of magnitude larger than the largest breakpoint density (1 per Mb) observed in this work using model M1 and 50-fold larger than the average breakpoint density over the complete genome (Table 1). As all our conclusions turn out to be valid for the two models, hence demonstrating that they are not subject to the biases mentioned above, all figures with breakpoint density estimates are presented for model M1 while the corresponding figures using model M2 are systematically provided as supplementary material in Additional file 1. Unless otherwise indicated, model M1 will therefore be implicitely assumed from now on.

**Table 1:** BPRs are preferentially intergenic.

Modelling	Average	Intergenes	Genes
M1	0.22 ± 0.01	0.28 ± 0.01	0.13 ± 0.01

M2	0.35 ± 0.01	0.43 ± 0.02	0.23 ± 0.02

Breakpoint density according to the two modellings of the BPR data set (see Results) estimated for intergenic and genic regions and averaged over all regions. Mean values and standard deviations are in breakpoint per Mb.

**Remark: **We mapped the positions of BPRs on the human genome while they do not necessarily concern evolutionary events that happened in the human lineage. In fact, the majority of them actually account for rearrangements in the rodent genomes (318/622, or 51%). For these events, a BPR on the human genome means that the rodent genome presents a breakage at the orthologous position. It therefore gives evidence for a susceptibility to breakage in the human region, that we may confront with evolutionarily stable genomic features such as isochores.

### BPRs are preferentially intergenic

Rearrangements are expected to be selected against if they break genes. It is the first basic assumption of the Intergenic Breakage Model [5,19]. Therefore BPRs are likewise expected to lie preferentially outside genes. To test whether genic content is avoided by BPRs, we compared the breakpoint density (see Methods) along genic and intergenic regions and averaged over the whole human genome (Table 1). The results correspond to the expectations: for the two modellings of the BPR data set, we observed that the intergenic (resp. genic) density is larger (resp. smaller) than the average genome density, with a twofold difference between intergenic and genic densities. We performed the same analysis for 1000 simulated BPR data sets with randomised positions. None of these simulations reproduced density differences as large as the observed, thus confirming that they are significantly larger than expected by a Random Breakage Model (*P <*0.001). These results agree with an intergenic breakpoint model, where breakpoints occur at random but are selected against in genic regions of a genome.

At first glance, the difference between intergenic and genic breakpoint densities suggests a rather weak effect of natural selection against breakage inside genes. However, when a BPR intersects a gene, that does not necessarily mean that the gene has been broken and become non functional. In fact, the major part of the genic breakpoint density is accounted by BPRs that overlap both genic and intergenic regions so that the breakpoints may have occurred outside the genes. Indeed, among the 265 BPRs that overlap a gene, only 57 are fully genic. The genic density can therefore simply be an effect of the lack of precision of our BPRs. The apparent breakage inside 57 genes may have different explanations, from bad annotation of the gene borders to the possible duplication of a broken region, that may or not have a relation to the rearrangement itself, leaving at least one functional copy of the concerned gene. For example, the human gene FAIM (Ensembl identifier ENSG00000158234) contains a BPR due to a rearrangement in the mouse lineage. A duplication in the mouse genome, probably linked to the rearrangement, yielded two annotated functional orthologs of the "broken" gene located on both sides of the rearranged region in the mouse genome (Ensembl identifiers ENSMUSG00000032463 and ENSMUSG00000079413). Hence, our genic breakpoint density is likely providing a somewhat misleading underestimation of the strength of natural selection. An accurate estimation of natural selection against breakages in genes would require an individual investigation of the evolutionary history of each gene/BPR pair, which is beyond the scope of the present study on the overall breakpoint distribution relatively to large-scale features of genomic organisation.

### Rearrangement breakpoints are over-represented in small intergenes

**Figure 2:**
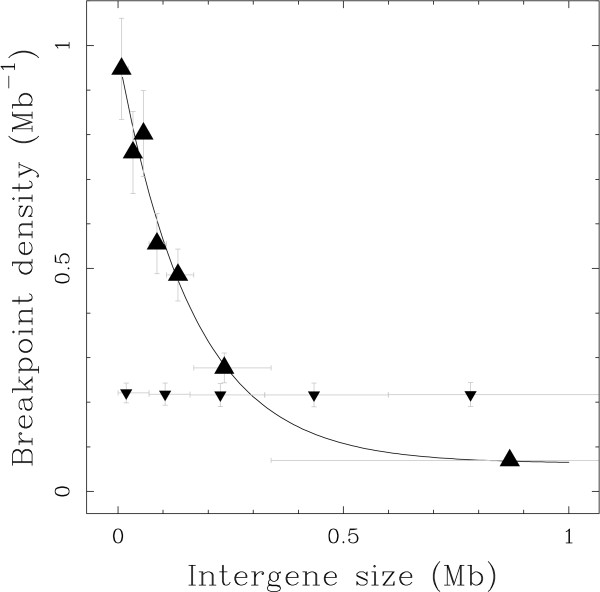
**Small intergenes present a high breakpoint density**. Intergenic breakpoint density (filled triangle, point up) estimated using model M1 versus intergene size. Mean intergenic breakpoint density (small filled triangle, point down) obtained as the average over 1000 simulated BPR data sets with randomised positions. Data points were obtained by (i) ordering intergenes according to their size, (ii) grouping them into classes of equal number of intergenic breakpoints and (iii) computing intergenic breakpoint density and average intergene size over each class. Vertical bars represent the standard deviations (see Methods); horizontal bars represent the ranges of intergene sizes over each class. The solid line corresponds to an exponential fit of the intergenic breakpoint density curve of equation: *d *= 0.063 + 0.92 exp(-*L/*165 kb) Mb^-1^.

Under a random model, or an intergenic model where genic regions are avoided, we expect the breakpoint density to be independent of the size of the intergenic regions. Moreover, selection may not only discard the breakage in genes, but also between genes and their regulatory regions, as hypothesised by Peng et al. [19], or in gene regulatory blocks, as hypothesised by Becker & Lenhard [5]. Under this "Intergenic Breakpoint Model", we thus expect the breakpoint density to grow with the size of the intergenic regions, since breakage in small intergenes, which necessarily lie close to a gene, should be avoided for their possible importance in gene regulation. Surprisingly, we observed that intergenic density actually negatively correlates with intergenic size (Fig. 2), the breakpoint density decreasing by more than one order of magnitude from 1 breakpoint per Mb in small intergenes (*L *< 20 kb) to 0.07 Mb^-1 ^for the largest intergenes (*L *> 300 kb). In comparison, we performed the same analysis for 1000 simulated BPR data sets with randomised positions and, as expected under an RBM, we did not observe any dependency of the intergenic breakpoint density with the intergenic size for these control data sets (Fig. 2). When using model M2 of the BPR data set, these results remained unchanged: breakpoint density is significantly higher in small intergenes than in large ones (~ tenfold; see Fig. S2 in Additional File 1).

**Figure 3:**
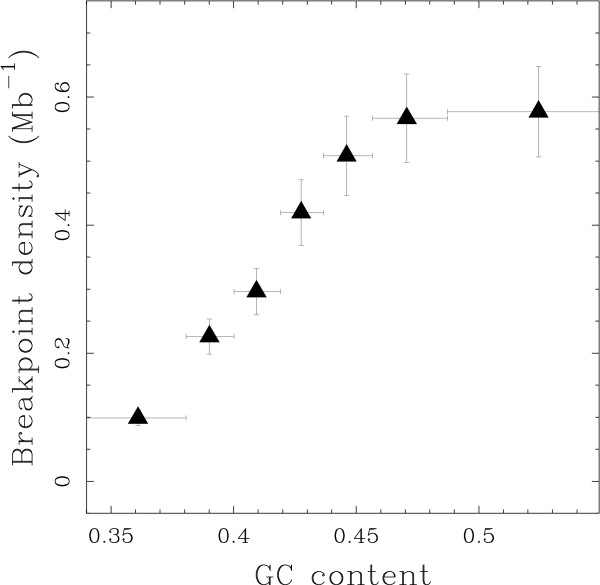
**Breakpoint density is higher in heavy isochors**. Intergenic breakpoint density estimated using model M1 versus GC content. Data points were obtained by (i) ordering 50 kb windows according to their GC content, (ii) grouping them into classes of equal number of intergenic breakpoints and (iii) computing intergenic breakpoint density and average GC content over each class. Vertical bars represent the standard deviations (see Methods); horizontal bars represent the ranges of GC content over each class.

In mammalian genomes, it is known that the gene distribution is highly heterogeneous and is linked to the organisation of the genome into isochores. Thus, small intergenes are concentrated inside regions of high GC-content, also referred to as heavy isochores [32-34]. Correspondingly, we found a higher breakpoint density in GC-rich regions of the genome (0.6 Mb^-1^) than in GC-poor regions (0.1 Mb^-1^) (Fig. 3; Fig. S3 in Additional File 1). These results suggest a model of evolution where the breakage probability depends on the genomic organisation. GC-rich regions, which exhibit small intergenes, seem to have a higher breakage probability along evolutionary time. In this study, isochores are observed in the human genome, whereas the breakages may have occurred in any mammalian lineage. However, isochores of mammalian genomes at this evolutionary scale are conserved [35-38]. This allows us to relate the breakage to an ancestral configuration of the genome, and thus to suppose that the breakage and the genomic organisation are contemporary.

Since intergene size correlates with GC content, it may be difficult to ascertain which of these two parameters best describes the positions of the BPRs. In order to disentangle the two, we analysed one while fixing the other. First, for five classes of GC content, we compared the median sizes of intergenes containing observed BPRs and randomised ones. For each class, we found that intergenes containing observed BPRs were two to three times smaller than intergenes corresponding to the randomised data set. These size differences are significant except for the class of highest GC content (*> *53%) due to its small size (Table 2). We then performed the reciprocal test to control for the effect of intergene size when correlating the BPR density with GC content. We did not find any significant difference in the median GC-content between BPRs and the simulated data set when considering classes of intergene size (see Table 1 in Additional File 1). It thus appears that the correlation between intergene size and BPR density cannot be fully explained by their respective correlation to the GC content, so that intergenic size comes out to be the appropriate parameter to describe susceptibility to breakage.

**Table 2:** BPRs lie in small intergenes.

	Median intergene size (kb)		
GC content (%)	BPRs	randomized regions	p-value
*GC *< 37	655 (n = 37)	1473 (n = 104)	4.7e-07

37 ≤ *GC *< 41	151 (n = 121)	514 (n = 147)	7.3e-11

41 ≤ *GC *< 46	84 (n = 172)	184 (n = 78)	6.5e-07

46 ≤ *GC *< 53	64 (n = 121)	90 (n = 45)	2.7e-02

*GC *≥ 53	19 (n = 25)	54 (n = 6)	0.90

Median intergene size of BPRs and randomised regions, classified in five classes of GC-content. For each class of GC content, the p-value of the non parametric Wilcoxon test between the BPRs and the randomized regions is given. GC content was measured inside each BPR region if the latter spans more than 50 kb, otherwise in a region of 50 kb centered on the BPR. Only regions whose central point lies inside an intergene are considered and the size of the latter is assigned to each region. The counts of regions in each class are indicated inside brackets.

## Discussion

We used a comparative genomics method based on the approach proposed by Lemaitre et al. [31], that allowed us to delineate a data set of 622 small regions of the human genome, the BPRs, that were modified by large structural mutations in one of the analysed mammalian genomes. On the one hand, we were able to quantitatively verify the hypothesis that rearrangements are selected against if they break genes (Table 1), thanks to the high resolution of the BPR data set (Fig. 1). On the other hand, contrary to what is expected under the Intergenic Breakage Model [5,19], the negative selection of rearrangements that break functional regions does not explain the observed heterogeneity of the BPR distribution. We showed that there is a significant preferential localisation of BPRs in small intergenes (Fig. 2). Correspondingly, BPR enrichment is also observed within GC-rich regions (heavy isochores) (Fig. 3), which are known to be gene dense and to be correlated with many other genomic features [32-34,39].

Our results are consistent with some previous observations in studies made at a lower resolution [22,40]. Also, in more specific studies, these observations were made by looking at restricted parts of some genomes, for example when human/chicken evolutionary breakpoints were compared [24] between two chicken chromosomes (11 and 28), when human-dog orthologs were studied in high GC regions [41], or a whole set of human/gibbon or human/cattle breakpoints [42,43]. In the latter case, the presence of many translocation breakpoints in gene rich regions was interpreted as positive selection acting on those genes. In the present work, we observed that GC content does not fully explain the decrease of breakpoint density with increasing intergene size. We propose that the heterogeneous distribution of breakpoints may be explained by the open state of the chromatin at the BPR positions. The regions of open chromatin are thought to be those of high transcriptional activity, early replication, accessibility to DNAse, and subject to DNA hypomethylation.

### Transcription

Our results suggest that evolutionary rearrangements tend to happen in regions of high transcriptional activity (in small intergenes, close to genes). Indeed, heavy isochores are regions of high gene density and thus of high transcriptional activity. In these regions, the DNA molecule has to be accessible to the proteins involved in transcription and the chromatin fiber is thought to be in a decondensed state (open chromatin) [44,45]. Because of this accessibility, such regions may also exhibit higher fragility to DNA double strand breaks, and in consequence to chromosomal rearrangements.

### Replication

**Figure 4:**
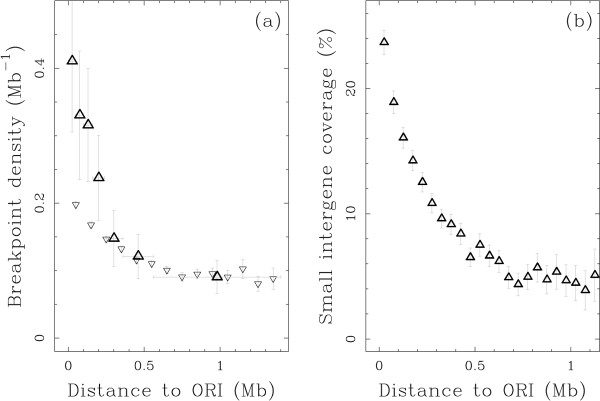
**Breakpoint density and small intergene coverage decrease with distance to putative replication origins**. (a) (open triangle, point up) Intergenic breakpoint density estimated using model M1 of the BPR data set versus the genomic distance to the closest putative origin located in the replication N-domains. (small open triangle, point down) Estimated intergenic breakpoint density in 100 kb windows along replication N-domains versus the distance to the closest ORI assuming that breakpoint density solely depends on the intergene size according to the fit of the breakpoint density versus intergenic size presented in Figure 2. Bars as in Figure 2. (b) Coverage of small intergenes (*L *≤ 150 kb) in 50 kb windows along replication N-domains versus the genomic distance to the closest putative replication origin. Small intergene coverage corresponds to the proportion of the sequence covered by a small intergene within the window of interest.

Regions of replication initiation are also likely to require an open chromatin state (an accessible DNA). Recently, Huvet et al. [46] discovered human genomic regions, called N-domains (see Methods), whose strand compositional asymmetry profiles are N-shaped as a footprint of the replication activity in the germline, and flanked by two putative replication origins (ORIs). They noted that these ORIs are at the heart of a remarkable gene organisation. Genes tend to lie close to the borders of N-domains, with a preferential co-orientation with the replication fork progression. Furthermore, broadly expressed genes are over-represented in the regions of the genome close to ORIs [46] and an analysis of high resolution replication timing data in relation to N-domains revealed that ORIs are likely to be early replicating and active in most tissues [47]. This led us to analyse the BPR locations with respect to the human ORIs. Within the 28.5% of the genome covered by N-domains, we observed a preference of BPRs to lie close to the putative replication origins (Fig. 4(a)) consistent with the observation of some increase of small intergene coverage (Fig. 4(b)). The intergenic breakpoint density decreases from 0.4 Mb^-1 ^close to the ORIs to less than 0.1 Mb^-1 ^in the middle of the N-domains. Moreover, intergenic breakpoint density close to the ORIs is larger than expected assuming that intergene size is the only factor governing the intergenic breakpoint density (0.2 Mb^-1^) (Fig. 4(a)) pointing to a specific effect due to the proximity to the ORIs. These observations suggest that evolutionary rearrangements tend to happen in regions of replication initiation.

### DNA hypomethylation

**Figure 5:**
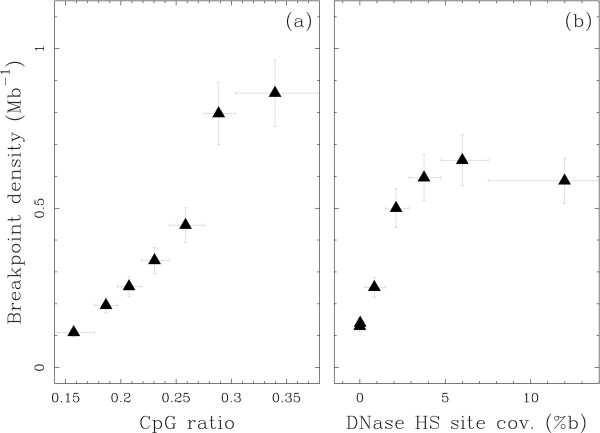
**Open chromatin regions present a high breakpoint density**. Intergenic breakpoint density estimated using model M1 versus (a) CpG ratio and (b) coverage by DNase I hypersensitive sites. Data points were obtained by (i) ordering 50 kb windows according to their CpG ratio (resp. Dnase I HS sites coverage), (ii) grouping them into classes of equal number of intergenic breakpoints and (iii) computing intergenic breakpoint density and average CpG ratio (resp. Dnase I HS sites coverage) over each class. Vertical bars represent the standard deviations (see Methods); horizontal bars represent the ranges of CpG ratio (resp. Dnase I HS sites coverage) over each class.

There are little available experimental data on the accessibility of genomic regions, especially in germline cells. Cytosine DNA methylation has been described as a mediator of gene silencing in repressed heterochromatin regions, while potentially active open chromatin regions would be essentially unmethylated [48,49]. High-throughput experiments are only starting to unravel genome-wide DNA methylation profiles, in particular in the complex genomes of vertebrates [50]. However, there is a genomic marker that is often used to estimate DNA methylation in the germline directly from the genomic sequence even though it was shown to present some dependency on the GC context [51]. It is based on the fact that CpG dinucleotides are highly mutable in the human genome in presence of methylation and it consists in computing the CpG observed/expected ratio (CpG o/e) [52,53]. To test the hypothesis that open chromatin is a determinant of the localisation of BPRs, we analysed the BPR density compared to the CpG o/e computed after removing known CpG islands from the analysis (CpG islands are known to localise often in the promoter regions of highly and widely expressed genes). A clear increase of the breakpoint density was observed, from 0.1 Mb^-1 ^in regions of lowest CpG o/e (high methylation level) to 0.9 Mb^-1 ^in regions with the largest CpG o/e (low methylation level) (Fig. 5(a)). Hence, high breakpoint density is found to be associated with the less methylated regions of the genome, likely to be the most accessible.

### DNAse sensitivity

Recently, experimental high resolution DNase I hypersensitive (HS) sites were made available as markers of open chromatin and high genomic activity across the complete human genome [54]. Di Filippo & Bernardi [55] have shown, using a smaller data set, that the DNase I HS sites density is greater in heavy isochores. Note that the Boyle et al. [54] data were obtained from primary human CD4^+^T cells and not from germline cells. Investigating breakpoint density versus DNase I HS sites coverage, we found an increase of the breakpoint density from 0.1 Mb^-1 ^in regions without any DNase I HS sites to 0.6 Mb^-1 ^in regions with the largest DNase I HS sites coverage (Fig. 5(b)). This comparison of breakpoint density to an experimental marker of genome accessibility provides additional support to the hypothesis that a major determinant of BPR localisation is open chromatin.

Such parameters all correlate with each other, so that it is difficult to disentangle them and find which are the true causal relationships. Nevertheless, we can control for the effects of some variables, and assess that GC content does not explain all the results. These observations are in agreement with our hypothesis that the regions of open chromatin make the genome more susceptible to evolutionary breakages, and may explain the surprising co-localisation of BPRs with small intergenes.

## Conclusion

In this paper, we analysed the relation between evolutionary breakpoint density and the organisation of the genome. This study shows that the susceptibility to breakage is highly heterogeneous along human chromosomes. Even though it is difficult to be definite on which parameter best describes the positions of BPRs since they all correlate with one another due to the organisation of the human genome into isochores, the results reported in this work reveal the importance of intergene size. The high breakpoint density in small intergenes can not be simply explained by the current model of structural genome evolution, namely the Intergenic Breakage Model, and we suggest a DNA accessibility-driven scenario, supported by data on methylation, transcriptional activity, replication initiation and experimental DNAse sensitivity, to explain this surprising result. If the proposition is correct, it would constitute the first evidence for a mechanistic explanation at a local scale (50 kb) without obvious relationship to natural selection whose influence is nevertheless clearly perceived on the systematically lower breakpoint density found in genic as compared to intergenic regions. Altogether our results enlighten the susceptibility of open chromatin regions to breakage as a third force to add to the evolutionary rearrangement description proposed by Becker & Lenhard [5] where positive selection is rare and negative selection protects some loci.

## Methods

### Sequence and annotation data

Sequence and annotation data were retrieved from the Genome Browsers of the University of California Santa Cruz (UCSC) [56] and of the Ensembl project [57]. The following releases of genome assemblies were used: human assembly of May 2004 (NCBI35 or hg17), chimpanzee assembly of March 2006 (panTro2.1), macaque assembly of February 2006 (MMUL1.0 or rheMac2), dog assembly of May 2005 (canFam2), mouse assembly of December 2005 (NCBIm35 or mm7), and rat assembly of December 2004 (RGSC 3.4 or rn4). Since chromosome Y was not available for all genomes, it was disregarded in all the analyses. In the same manner, all unsequenced region (e.g. centromeres) were not included in the analysis. As human gene coordinates, we used the UCSC Known Genes table. When several genes presenting the same orientation overlapped, they were merged into one gene whose coordinates corresponded to the union of all the overlapping gene coordinates. This resulted in 20349 distinct genes over the 22 human autosomes and chromosome X.

GC content was computed over the native sequence. We checked that the results remained qualitatively similar when considering the GC content computed over the repeat-masked sequence or when masking CpG islands as annotated in the UCSC database. CpG observed/expected ratio (CpG o/e) was computed as , where *n*_*C*_, *n*_*G *_and *n*_*CpG *_are the number of C, G and dinucleotide CG counted along the sequence, *L *is the number of non-masked nucleotides of the sequence and *l *the number of masked nucleotide gaps plus one, i.e. *L *- *l *is the number of dinucleotide sites. The CpG o/e was computed over the sequence where annotated CpG islands were masked. We checked that the results remained qualitatively similar when considering the CpG o/e computed over the native sequence or over the repeat-masked sequence.

The 95 723 experimental DNase I hypersensitive (HS) site data (UCSC "dukeDnaseCd4Sites" track) corresponds to genome-wide DNase I HS sites as determined for human CD4+ T-cells using DNase-sequencing and DNase-chip by Boyle et al. [54].

### Precise detection of rearrangement breakpoint regions

In order to analyse the positions of evolutionary breakpoints relatively to small scale features of the genome such as genes or replication domains, we constructed a high resolution database of BPR coordinates along the human genome using a novel methodology, henceforth denoted by CASSIS, described in [31]. This method precisely locates BPRs on a reference genome by comparison with the genome of a related species. Contrary to previous methods which search for synteny blocks and simply return what remains in the genome as BPRs, the framework of CASSIS consists in building synteny blocks from orthologous markers and in further investigating the BPRs themselves in order to refine their coordinates. By aligning each breakpoint sequence on the reference genome against its specific orthologous sequences in the other species, CASSIS looks for weak similarities inside the BPRs, thus extending the synteny blocks and narrowing the BPRs [31]. Using this method, the human genome was compared to the following five eutherian mammals which have a sequenced and assembled genome: chimpanzee, macaque, mouse, rat and dog. For each human/non-human pairwise comparison, we used the one-to-one orthologous genes available from the Ensembl genome browser [57] as the orthologous markers to build the initial synteny blocks. For the purpose of this paper, we extended the method of Lemaitre et al. [31] by combining the coordinates of overlapping BPRs obtained from each of the pairwise comparisons to build the final set of BPR coordinates. We identified which BPR common to several genome pairs were likely to account for the same rearrangement event by building a graph *G *where vertices are the BPRs mapped on the human genome, and edges join every two intersecting BPRs. A group *S *of species is considered *monophyletic *in an unrooted phylogenetic tree if every leaf of the smallest sub-tree containing all species from *S *are in *S*. For every connected component of the graph *G*, we tested if:

• there exists a unique common intersection to all BPRs;

• the non-human species involved in the component are monophyletic.

If both conditions are satisfied, then all the BPRs in the component may account for a single rearrangement event. If one condition is not satisfied, then at least two events are necessary to explain the component. In the first case, a single BPR is constructed from the component by taking the common intersection of all the regions in the component. In the second case, a single BPR is constructed from the component by taking the union of all regions involved in the component (see a full description of the method in Additional File 1). We thus obtained 622 BPRs. The size distribution of the BPRs has a mean value of 104 kb, a standard deviation of 252 kb and a median size of 26.6 kb (Fig. 1). The coordinates of BPRs on the human genome along with their evolutionary branch assignment are provided in Additional File 2, together with a high level graphical representation of their distribution along the human chromosomes (Additional File 3).

### Estimating breakpoint density

A genomic subset *GS *is the union of non-overlapping intervals *GS*_*j *_of size *L*(*GS*_*j*_) along the human genome. The BPRs constitute a particular genomic subset defined by intervals *B*_*i *_of size *L*(*B*_*i*_). In order to be able to take into account the fact that each BPR may actually correspond to several independent breakage events, we associated a number *n*_*i *_of breakpoints to every *B*_*i *_(1 in the M1 model of the BPR data set). Furthermore, since we lacked information on the exact location of breakpoints within the BPRs, we associated a uniform breakpoint density *d*(*B*_*i*_) = *n*_*i*_/*L*(*B*_*i*_) to each *B*_*i*_. Then, the expectation of the number of breakpoints over an interval of interest *GS*_*j *_is *E*(*n*_*j*_) = Σ_*i*_*d*(*B*_*i*_)*L*(*B*_*i *_∩ *GS*_*j*_). The breakpoint density *d*_*GS *_for the set of intervals *GS*_*j *_is defined by the following expectation value:



For instance, if we compute the breakpoint density over genic regions of the genome on one hand (the genic breakpoint density) and over intergenic regions on the other hand (the intergenic breakpoint density), a BPR spanning both genic and intergenic regions will be counted both times but with a weight proportional to its overlap with, respectively, the genic and the intergenic regions.

Assuming the RBM is applicable when considering the distribution of breakpoints along the set of intervals *GS*_*j*_, the estimated number of breakpoints over these intervals corresponds to the expectation of a binomial distribution for a series of *L*({*GS*_*j*_}) = Σ_*j *_*L*(*GS*_*j*_) trials of probability *d*_*GS*_. In this framework, an a priori standard deviation for *d*_*GS *_reads .

### Randomisation of BPR positions

As control experiments, we studied the breakpoint density when the set of BPRs is chosen at random along the genome, respecting the size distribution of the observed set of BPRs. A BPR data set with randomised positions was constructed by randomly taking each BPR one by one and randomly re-positioning it along the complete genome avoiding overlap with a previously re-positioned BPR or with an assembly gap of size *> *200 kb. One thousand BPR data sets with randomised positions were generated. When averaging breakpoint density estimations *d*_*s *_over these 1000 BPR data sets, we also computed the empirical standard deviation, comparable to the a priori standard deviation used for the observed BPR data sets, as:  where  is the arithmetic mean of *d*_*s*_.

### Replication domains and putative replication origins

The detection of human replication *N-domains *is based on the mammalian replicon model [58,59]. This model with fixed replication origins and randomly distributed termination sites, imposes an N-shaped profile for the nucleotide compositional strand asymmetry () between two successive fixed replication origins. Using the wavelet transform as a multi-scale (the distance between origins is highly variable) shape detector, Huvet et al. [46] developed a very efficient segmentation strategy of the human genome into candidate replication domains where the skew *S *displays the characteristic N-shaped pattern. Note that this segmentation strategy is less efficient in GC rich regions of the genome. Indeed, the smaller N-domain size and the high gene density in these regions make it difficult to distinguish replication-related from transcription-related strand asymmetry [46].

The coordinates of the 678 human replication N-domains were obtained directly from the authors [46]. The 1060 N-domain borders (in 296 cases, a border is shared by two domains) were considered as putative replication origins (ORIs). Within the 22 human autosomes and chromosome X, the N-domains cover 28.5% of the sequenced genome length and 17% of the genes (3431 gene starts are in an N-domain).

## Authors' contributions

The conception of the study and writing of the manuscript was performed conjointly by all the authors. CL, LZ, ET and BA performed the statistical analyses. All the authors read and approved the final manuscript.

## Supplementary Material

Additional file 1 **Additional information on the mapping of BPRs on the human genome – Supplementary figures and tables**. We present complementary details on the detection of BPRs and propose an algorithm to assign breakpoints to a branch of the phylogenetic tree. This file also contains the figures and table presented in the main text when estimating breakpoint density using the control model M2. Click here for file

Additional file 2 **BPR localisation and phylogenetic assignment**. This file consist of a complete list of the BPRs analysed along with their phylogenetic assignment (See Additional File 1). Click here for file

Additional file 3 **Representation of BPRs along human chromosomes**. For each chromosome, we display BPRs in blue, replication N-domains in green and a profile of the native GC content computed in 100 kb windows in pink. Click here for file
