# Supplementary material for: Analysis of fine-scale mammalian evolutionary breakpoints provides new insight into their relation to genome organisation
Source: BMC Genomics. 2009 Jul 24;10:335. doi: 10.1186/1471-2164-10-335 (PMC2722678; doi:10.1186/1471-2164-10-335)
Supplement: Additional file 1 — Additional information on the mapping of BPRs on the human genome – Supplementary figures and tables. We present complementary details on the detection of BPRs and propose an algorithm to assign breakpoints to a branch of the phylogenetic tree. This file also contains the figures and table presented in the main text when estimating breakpoint density using the control model M2. [file 1471-2164-10-335-S1.pdf]

Additional File 1 for

Analysis of fine-scale mammalian evolutionary breakpoints provides new insight into their relation to genome organisation

Claire Lemaitre[1,2,3,4,*], Lamia Zaghloul[2,5,*], Marie-France Sagot[2,3,4], Christian Gautier[2,3,4], Alain Arneodo[2,5], Eric Tannier[2,3,4,†] and Benjamin Audit[2,5,‡]

[1] *Université de Bordeaux, Centre de Bioinformatique - Génomique Fonctionnelle Bordeaux, F-33000 Bordeaux, France;*
[2] *Université de Lyon, F-69000 Lyon, France;*
[3] *Laboratoire Biométrie et Biologie Evolutive, CNRS, Université Lyon 1, F-69100 Villeurbanne, France;*
[4] *Équipe BAMBOO, INRIA Rhône-Alpes, 655 avenue de l'Europe, F-38330 Montbonnot Saint-Martin, France;*
[5] *Laboratoire Joliot-Curie et Laboratoire de Physique, CNRS, Ecole Normale Supérieure de Lyon, F-69007 Lyon, France;*

[*] *These authors contributed equally to this work.*
[†] *Corresponding author. Email Eric.Tannier@inria.fr; Fax 33-4-72-43-13-88.*
[‡] *Corresponding author. Email Benjamin.Audit@ens-lyon.fr; Fax 33-4-72-72-80-80.*

Mapping of all pairwise genome comparisons on the human genome

The result of the pairwise comparisons (human genome versus a mammalian genome) is a set of coordinates, on the human genome, of the breakpoints. Some of them may intersect when they arise from the comparison with different species. The cause of an intersection of two breakpoints is either the fact there has been a unique event in the human lineage after the speciation with the closest species, or the occurence of two independant breakages in two regions that are too close to disentangle them. In the first case, the breakage occured in the intersection of the two regions, and in the second there were at least two events in the union of the two regions. When a set of (possibly more than two) breakpoints intersect, then we explain all of them with a single event if

- all of them have a common intersection;
- the set of non-human species which are involved in the set of breakages is monophyletic in the unrooted mammalian phylogenetic tree.

Indeed these two conditions are clearly necessary to explain all breakpoints with a single event. They are not fully sufficient, as even if they are satisfied, there is no proof that there has been a single event, all the more since this method is sensitive to the detection of small synteny blocks, and its specificity has not been tested.

A BPR is either the intersection or the union of the set of intersecting pairwise breakpoints. In the first case, it is possible to assign the breakage event to a branch of the phylogenetic tree. We made no use of this information, but added it to the data (Additional File 2). In the second case, we called the BPR "reused", as at least two breakages have occurred in this region in mammalian history. The assignments should be taken with caution because it is possible that the method lacks specificity and this has not been evaluated.

We found that out of the 622 BPRs, 40 at least are used more than once.

Supplementary Figures

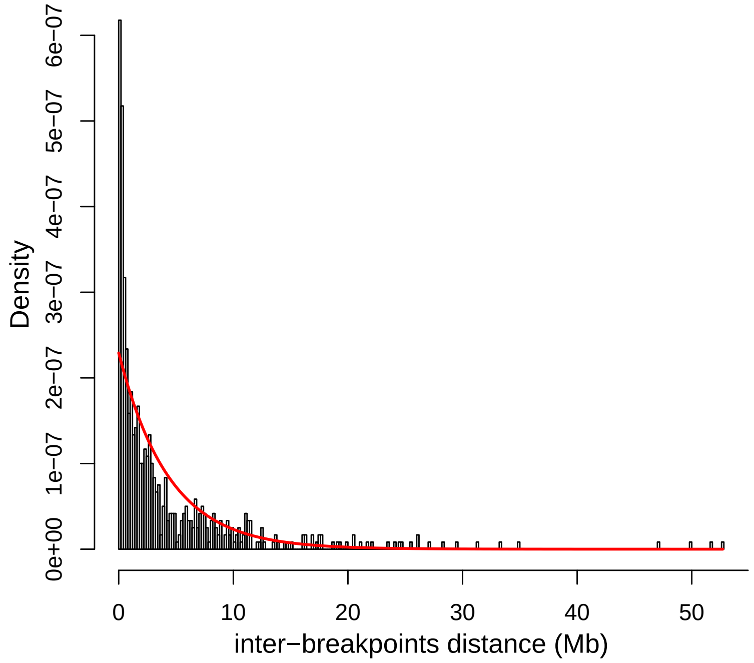

Figure S1: Distribution of inter-breakpoints distances fitted to an exponential distribution (red line) of parameter $\lambda = 2.29 \times 10^{-07}$. It does not fit an exponential distribution due to an excess of small distances (the Kolmogorov-Smirnov goodness-of-fit test between the two distributions gives a p-value $< 10^{-16}$). This indicates some clustering of the breakpoints along the human chromosomes and rebuts the Random Breakage Model.

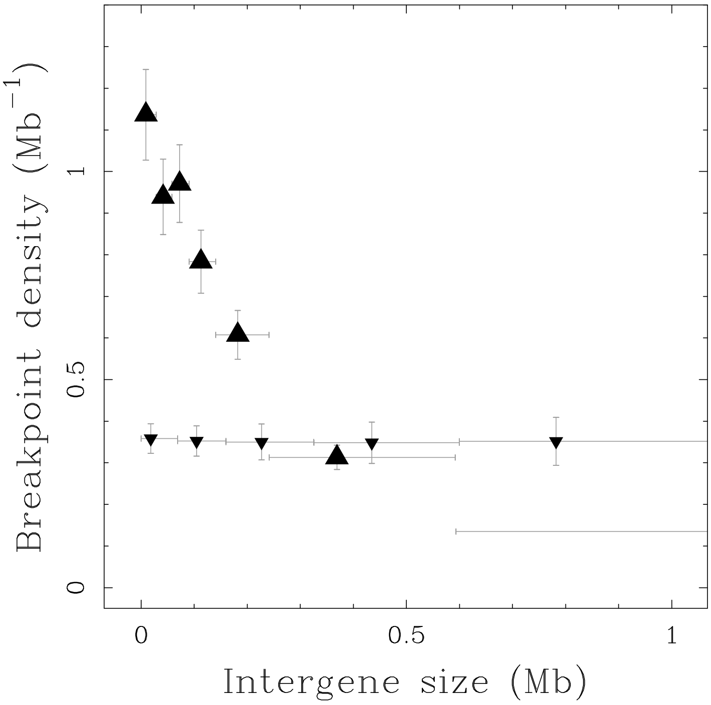

Figure S2: Intergenic breakpoint density (filled triangle, point up) estimated using model M2 of the BPR data set versus intergene size. Mean intergenic breakpoint density (small filled triangle, point down) obtained as the average over 1000 simulated BPR data sets with randomised positions. Data points were obtained by (i) ordering intergenes according to their size, (ii) grouping them into classes of equal number of intergenic breakpoints and (iii) computing intergenic breakpoint density and average intergene size over each class. Vertical bars represent the standard deviations; horizontal bars represent the ranges of intergene sizes over each class.

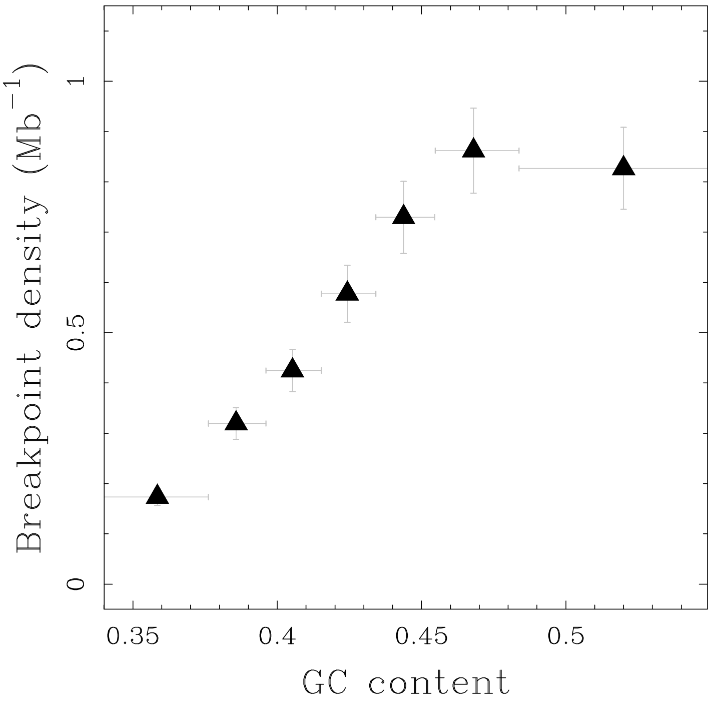

Figure S3: Intergenic breakpoint density estimated using model M2 versus GC content. Data points were obtained by (i) ordering 50 kb windows according to their GC content, (ii) grouping them into classes of equal number of intergenic breakpoints and (iii) computing intergenic breakpoint density and average GC content over each class. Vertical bars represent the standard deviations; horizontal bars represent the ranges of GC content over each class.

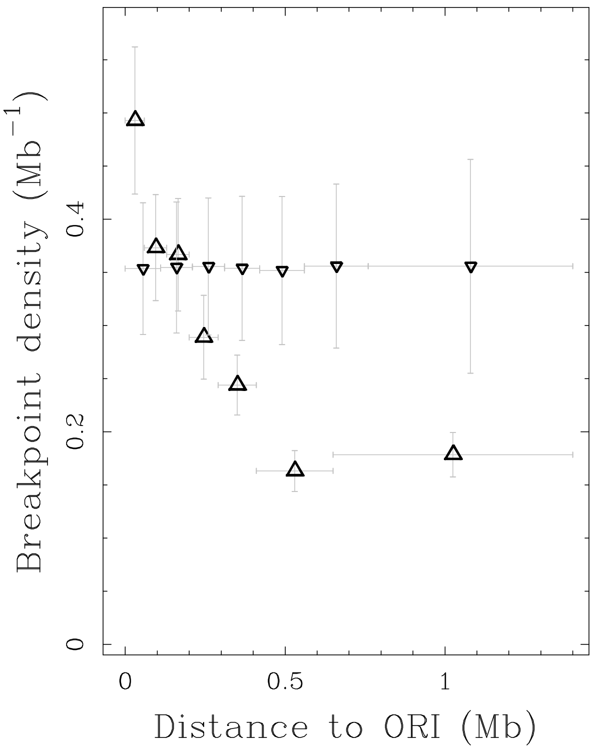

Figure S4: Intergenic breakpoint density (open triangle, point up) estimated using model M2 of the BPR data set versus the genomic distance to the closest putative origin located in the replication N-domains. Mean intergenic breakpoint density (small open triangle, point down) obtained as the average over 1000 simulated BPR data sets with randomised positions.

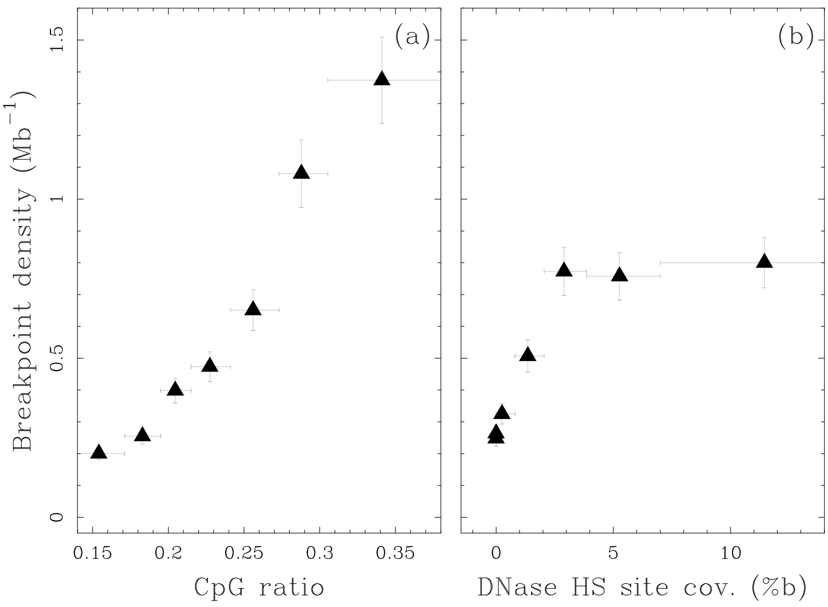

Figure S5: Intergenic breakpoint density estimated using model M2 versus (a) CpG ratio and (b) DNase I hypersensitive sites coverage. Data points were obtained by (i) ordering 50 kb windows according to their CpG ratio (resp. Dnase I HS sites coverage), (ii) grouping them into classes of equal number of intergenic breakpoints and (iii) computing intergenic breakpoint density and average CpG ratio (resp. Dnase I HS sites coverage) over each class. Vertical bars represent the standard deviations (see Methods); horizontal bars represent the ranges of CpG ratio (resp. Dnase I HS sites coverage) over each class.

	Median GC content (%)		
intergene size S (Kb)	BPRs	randomised regions	p-value
$S < 50$	45.3 (n=144)	45.5 (n=37)	0.76
$50 \leq S < 100$	43.5 (n=120)	42.7 (n=39)	0.98
$100 \leq S < 200$	43.2 (n=94)	42.8 (n=42)	0.14
$200 \leq S < 500$	40.9 (n=68)	40.3 (n=76)	0.54
$S \geq 500$	37.6 (n=50)	37.1 (n=186)	0.19

Table 1: Median GC content of BPRs and randomised regions, classified in five classes of intergene size. For each class of intergene size, the p-value of the non parametric Wilcoxon test between the BPRs and the randomised regions is given. GC content was measured inside each BPR region if the latter spans more than 50 kb, otherwise in a region of 50 kb centered on the BPR. Only regions whose central point lies inside an intergene are considered and the size of the latter is assigned to each region. The number of regions in each class are indicated inside brackets.
