# Supplementary material for: Analysis of fine-scale mammalian evolutionary breakpoints provides new insight into their relation to genome organisation
Source: BMC Genomics. 2009 Jul 24;10:335. doi: 10.1186/1471-2164-10-335 (PMC2722678; doi:10.1186/1471-2164-10-335)
Supplement: Additional file 3 — Representation of BPRs along human chromosomes. For each chromosome, we display BPRs in blue, replication N-domains in green and a profile of the native GC content computed in 100 kb windows in pink. [file 1471-2164-10-335-S3.pdf]

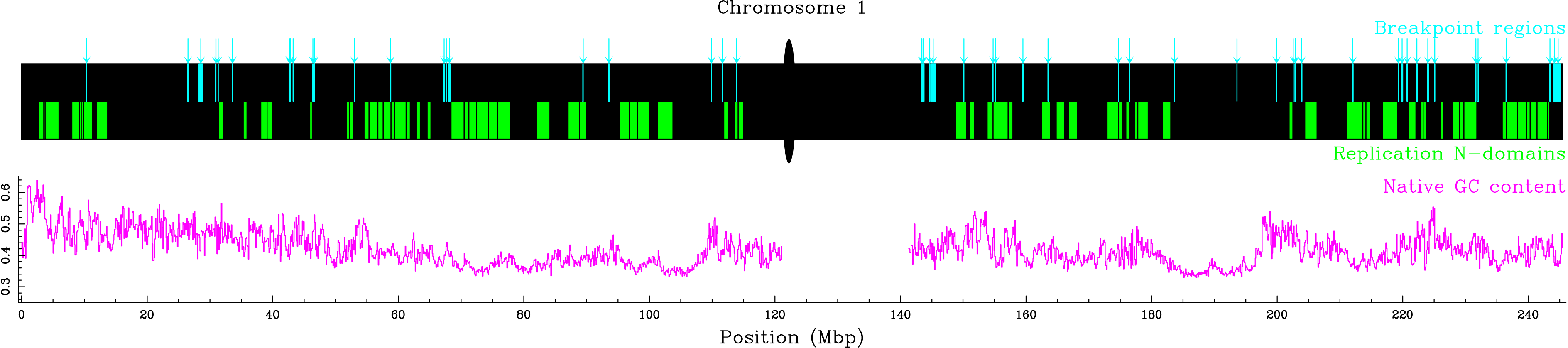

Chromosome 1
Breakpoint regions
Replication N–domains
Native GC content
0.3
0.4
0.5
0.6
0
20
40
60
80
100
120
140
160
180
200
220
240
Position (Mbp)

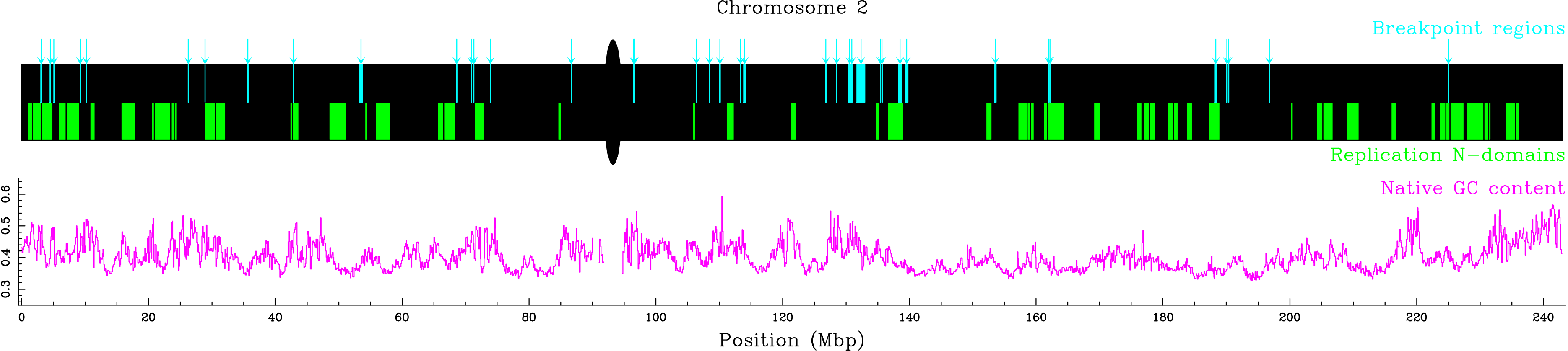

Chromosome 2
Breakpoint regions
Replication N-domains
Native GC content
0.3
0.4
0.5
0.6
0
20
40
60
80
100
120
140
160
180
200
220
240
Position (Mbp)

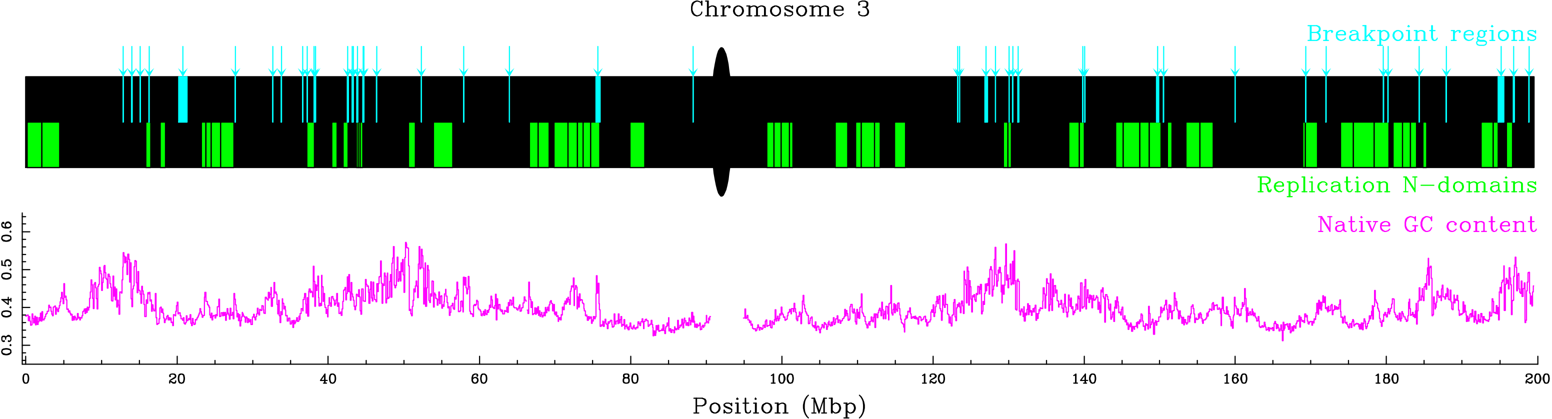

Chromosome 3
Breakpoint regions
Replication N-domains
Native GC content
0.3
0.4
0.5
0.6
0
20
40
60
80
100
120
140
160
180
200
Position (Mbp)

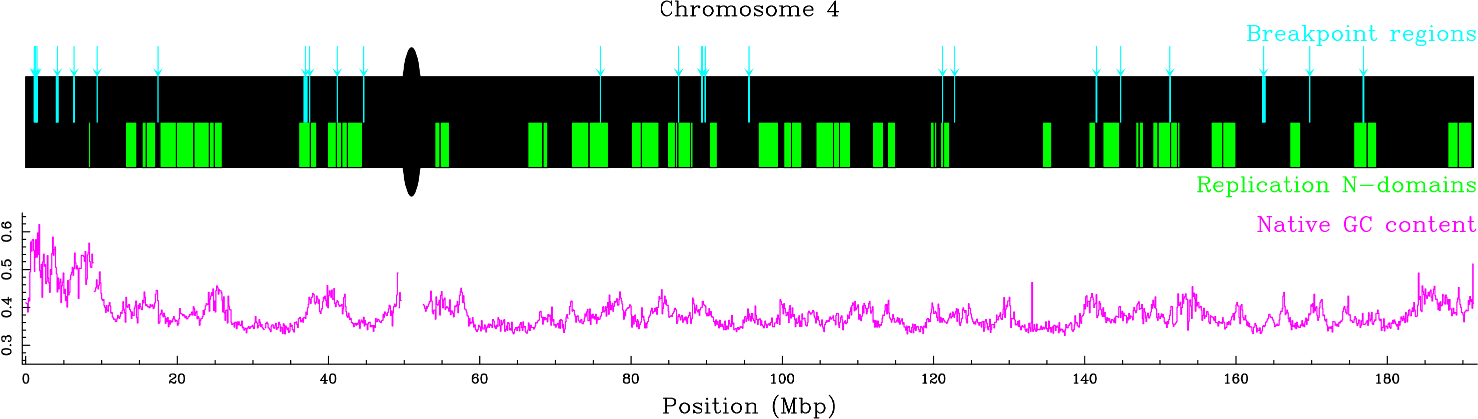
Chromosome 4
Breakpoint regions
Replication N−domains
Native GC content
0.3
0.4
0.5
0.6
0
20
40
60
80
100
120
140
160
180
Position (Mbp)

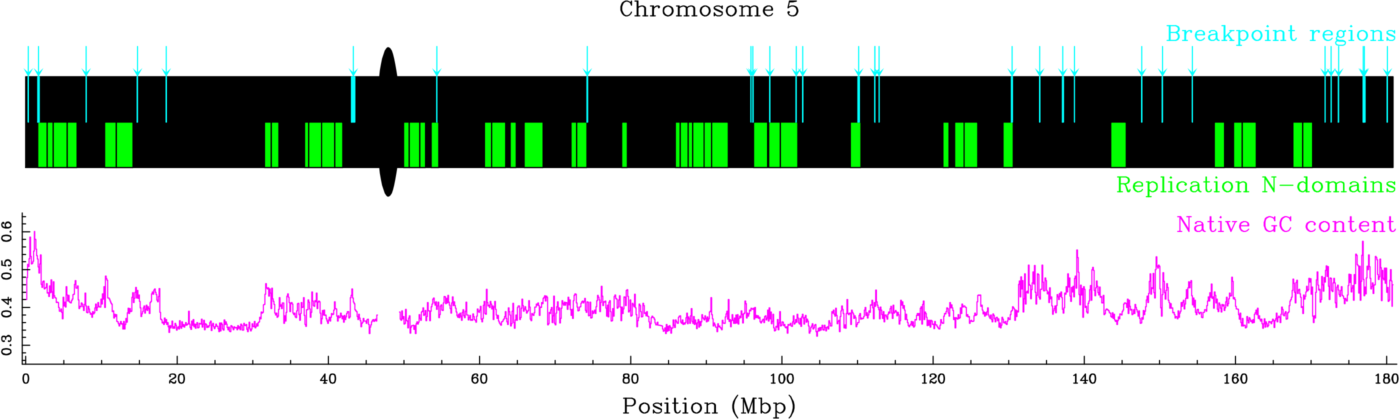

Chromosome 5
Breakpoint regions
Replication N-domains
Native GC content
0.3
0.4
0.5
0.6
0
20
40
60
80
100
120
140
160
180
Position (Mbp)

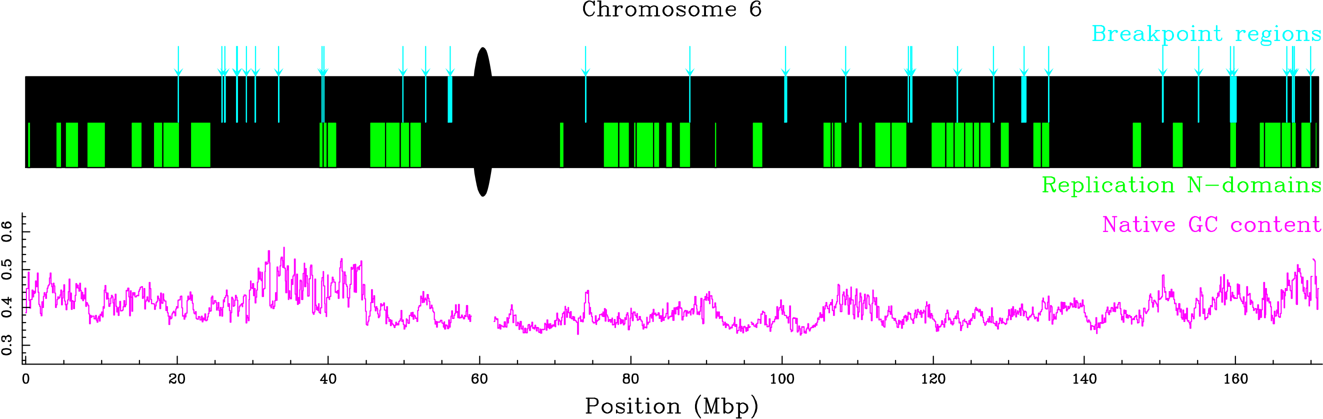

Chromosome 6
Breakpoint regions
Replication N-domains
Native GC content
0.3
0.4
0.5
0.6
0
20
40
60
80
100
120
140
160
Position (Mbp)

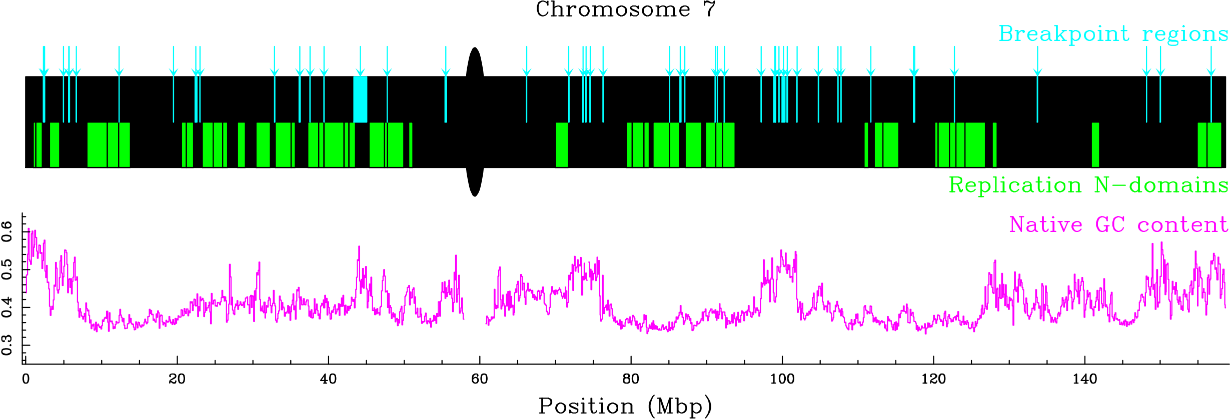

Chromosome 7
Breakpoint regions
Replication N-domains
Native GC content
0.3
0.4
0.5
0.6
0
20
40
60
80
100
120
140
Position (Mbp)

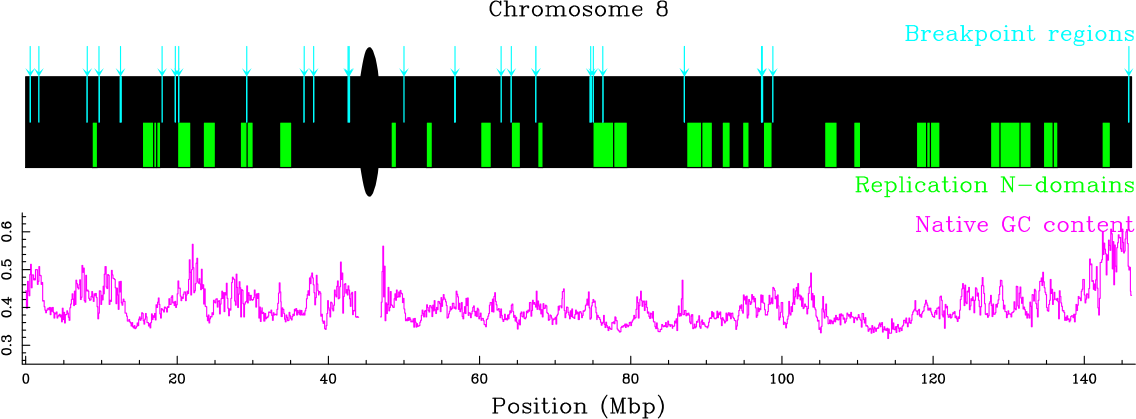

Chromosome 8
Breakpoint regions
Replication N−domains
Native GC content
0.3
0.4
0.5
0.6
0
20
40
60
80
100
120
140
Position (Mbp)

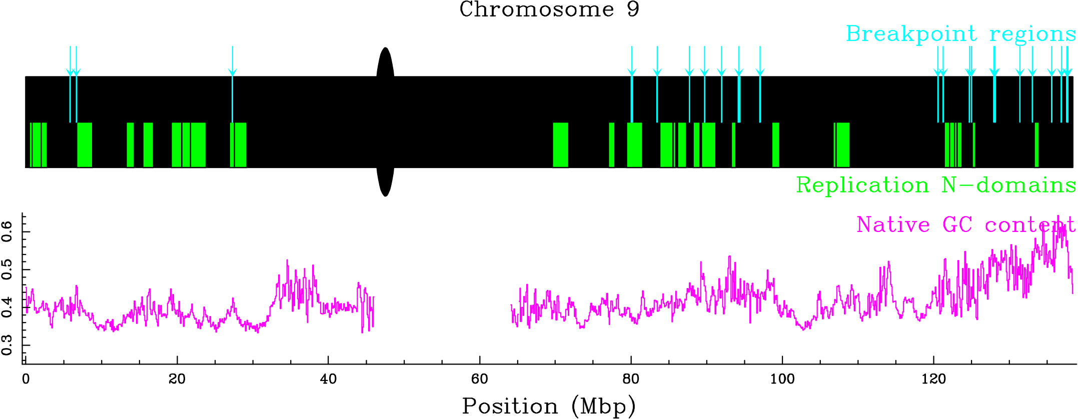

Chromosome 9
Breakpoint regions
Replication N-domains
Native GC content
0.3
0.4
0.5
0.6
0
20
40
60
80
100
120
Position (Mbp)

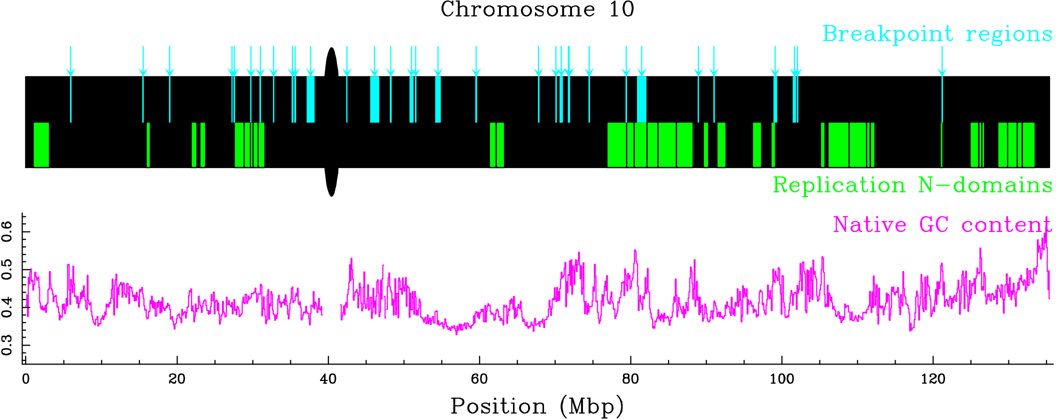
Chromosome 10
Breakpoint regions
Replication N–domains
Native GC content
0.3
0.4
0.5
0.6
0
20
40
60
80
100
120
Position (Mbp)

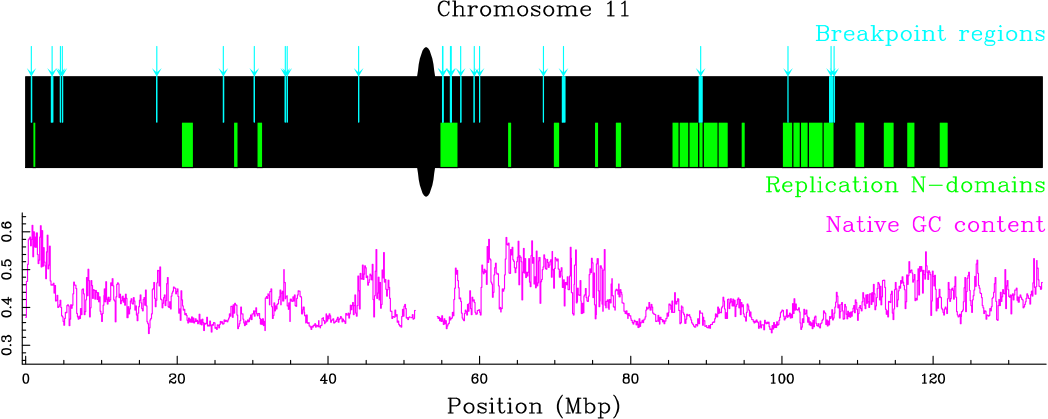

Chromosome 11
Breakpoint regions
Replication N−domains
Native GC content
0.3
0.4
0.5
0.6
0
20
40
60
80
100
120
Position (Mbp)

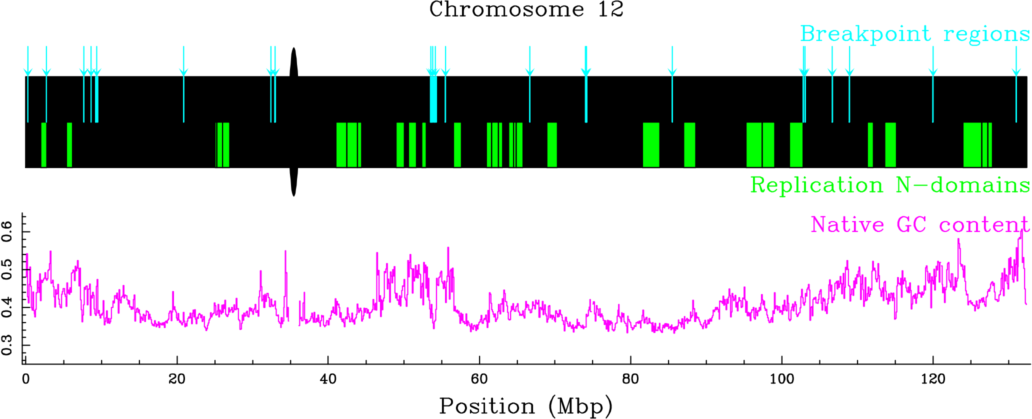

Chromosome 12
Breakpoint regions
Replication N-domains
Native GC content
0.3
0.4
0.5
0.6
0
20
40
60
80
100
120
Position (Mbp)

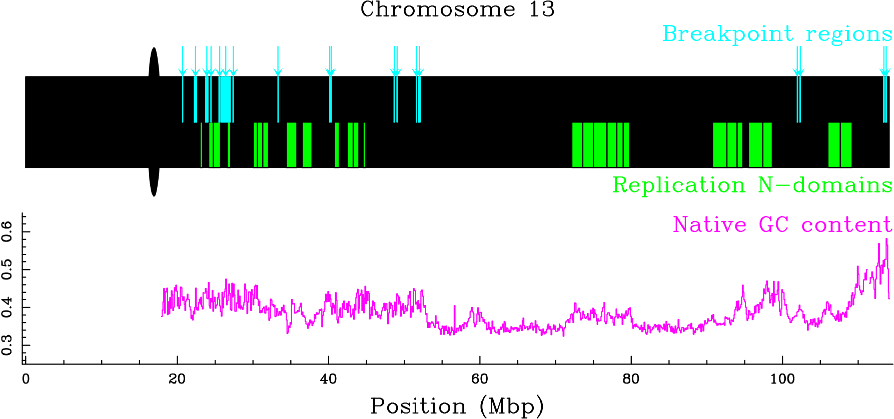

Chromosome 13
Breakpoint regions
Replication N-domains
Native GC content
0.3
0.4
0.5
0.6
0
20
40
60
80
100
Position (Mbp)

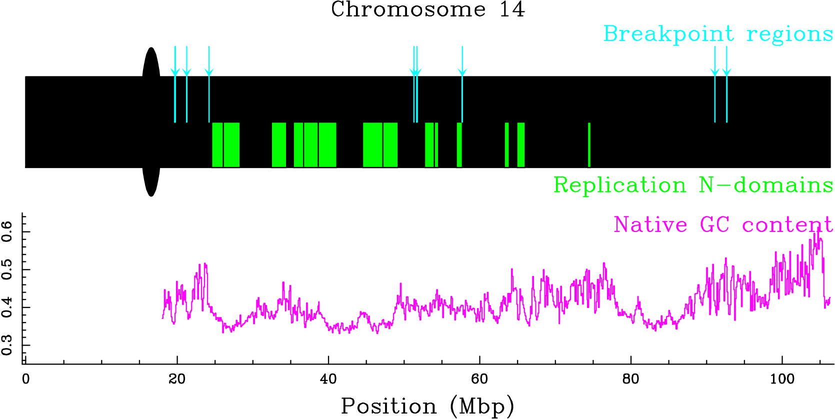

Chromosome 14
Breakpoint regions
Replication N−domains
Native GC content
0.3
0.4
0.5
0.6
0
20
40
60
80
100
Position (Mbp)

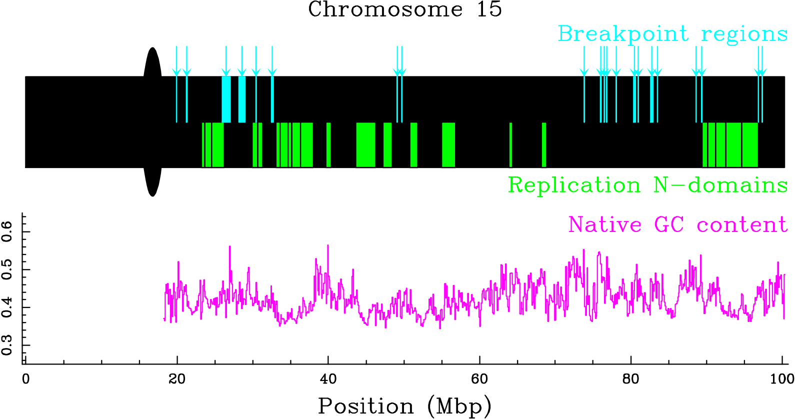

Chromosome 15
Breakpoint regions
Replication N−domains
Native GC content
0.3
0.4
0.5
0.6
0
20
40
60
80
100
Position (Mbp)

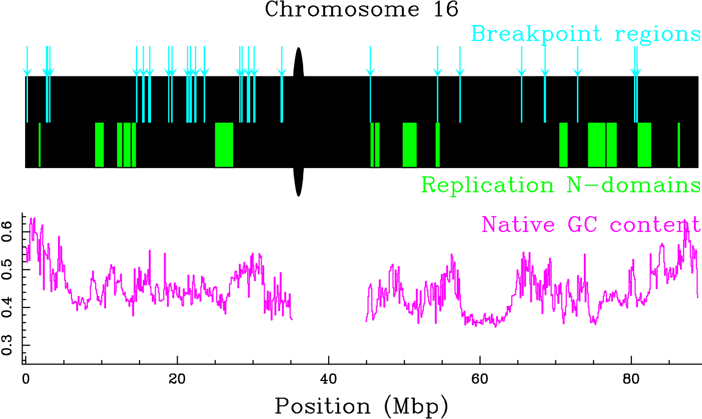

Chromosome 16
Breakpoint regions
Replication N-domains
Native GC content
0.3
0.4
0.5
0.6
0
20
40
60
80
Position (Mbp)

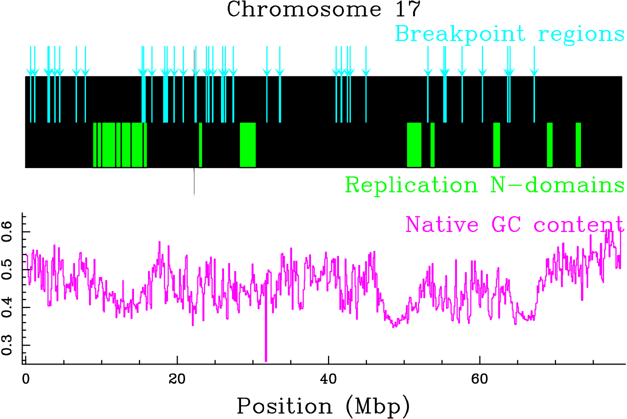

Chromosome 17
Breakpoint regions
Replication N-domains
Native GC content
0.3
0.4
0.5
0.6
0
20
40
60
Position (Mbp)

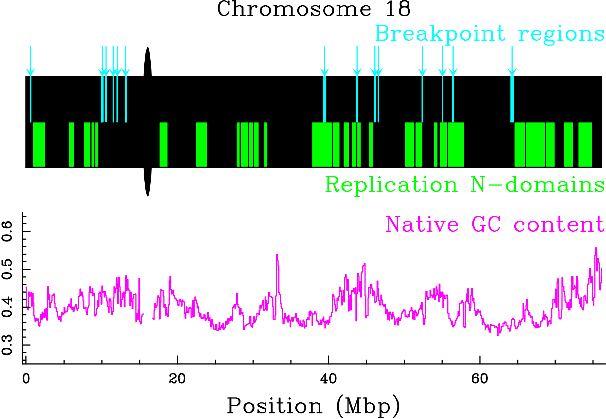

Chromosome 18
Breakpoint regions
Replication N-domains
Native GC content
0.3
0.4
0.5
0.6
0
20
40
60
Position (Mbp)

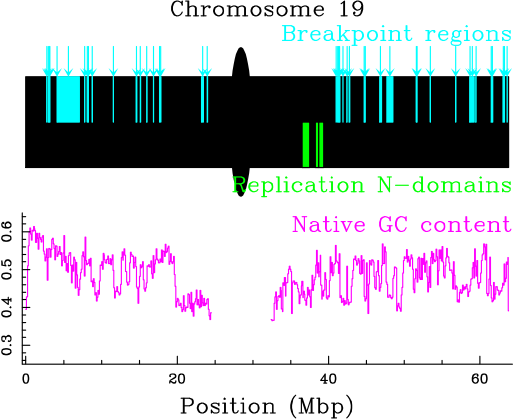

Chromosome 19
Breakpoint regions
Replication N-domains
Native GC content
0.6
0.5
0.4
0.3
0
20
40
60
Position (Mbp)

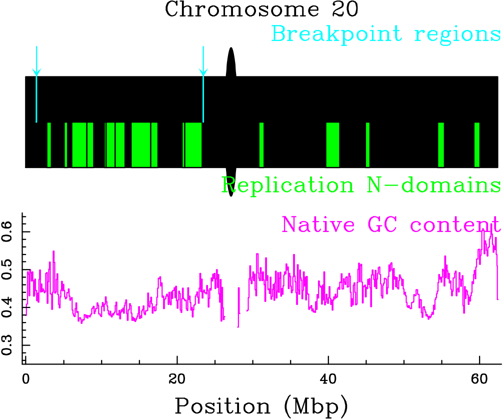

Chromosome 20
Breakpoint regions
Replication N-domains
Native GC content
0.3
0.4
0.5
0.6
0
20
40
60
Position (Mbp)

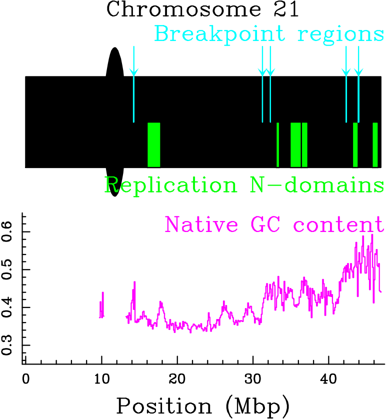
Chromosome 21
Breakpoint regions
Replication N-domains
Native GC content
0.3
0.4
0.5
0.6
0
10
20
30
40
Position (Mbp)

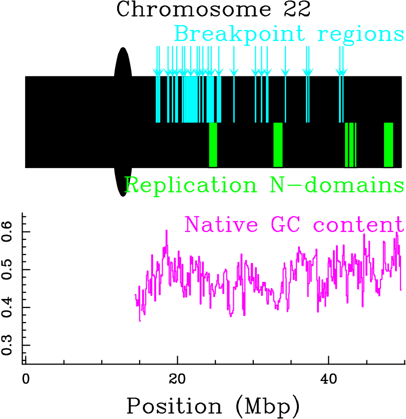

Chromosome 22
Breakpoint regions
Replication N-domains
Native GC content
0.3
0.4
0.5
0.6
0
20
40
Position (Mbp)

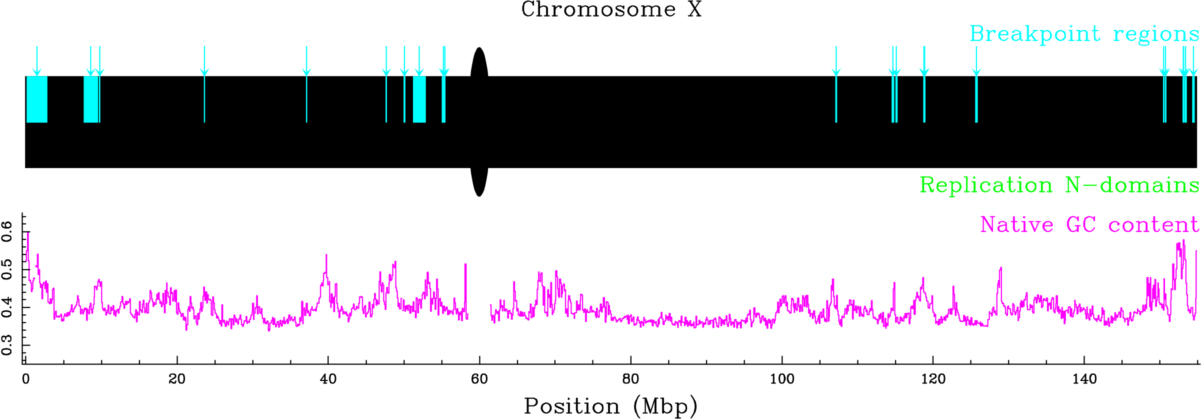

Chromosome X
Breakpoint regions
Replication N-domains
Native GC content
0.3
0.4
0.5
0.6
0
20
40
60
80
100
120
140
Position (Mbp)
